# Evolution of treatment patterns and survival outcomes in patients with advanced non-small cell lung cancer treated at Frankfurt University Hospital in 2012–2018

**DOI:** 10.1186/s12890-022-02288-1

**Published:** 2023-01-13

**Authors:** Andrea Wolf, Jan A. Stratmann, Shabnam Shaid, Nicolas Niklas, Alan Calleja, Harveen Ubhi, Robin Munro, Daniela Waldenberger, Robert Carroll, Melinda J. Daumont, John R. Penrod, Laure Lacoin, Gernot Rohde

**Affiliations:** 1 University Cancer Center, University Hospital Frankfurt, Theodor-Stern-Kai 7, 60590 Frankfurt am Main, Germany; 2 Department of Medicine, Hematology/Oncology, Goethe University, Frankfurt am Main, Germany; 3 Real World Solutions, IQVIA, Frankfurt, Germany; 4 Real World Solutions, IQVIA, London, UK; 5 Medical Oncology, Bristol Myers Squibb GmbH & Co. KGaA, Munich, Germany; 6 Centre for Observational Research and Data Sciences, Bristol Myers Squibb, Uxbridge, UK; 7 Worldwide Health Economics and Outcomes Research, Bristol Myers Squibb, Braine-l’Alleud, Belgium; 8 Worldwide Health Economics and Outcomes Research, Bristol Myers Squibb, Princeton, NJ USA; 9 Epi-Fit, Bordeaux, France; 10 Medical Clinic I, Department of Respiratory Medicine, University Hospital Frankfurt, Frankfurt am Main, Germany

**Keywords:** Immune checkpoint inhibitors, Immunotherapy, Non-small cell lung cancer, Overall survival, Real-world evidence

## Abstract

**Background:**

Immune checkpoint inhibitors (ICIs) have improved outcomes for patients with advanced non-small cell lung cancer (NSCLC) versus chemotherapy in clinical trials. In Germany, ICIs have been used clinically since 2015 for patients with advanced/metastatic NSCLC without epidermal growth factor receptor (*EGFR*)/anaplastic lymphoma kinase (*ALK*) aberrations. As part of I-O Optimise, a multinational research program utilizing real-world data on thoracic malignancies, we describe real-world treatment patterns and survival following reimbursement of ICIs for advanced NSCLC in Germany.

**Methods:**

This retrospective cohort study included patients with locally advanced/metastatic NSCLC without known *EGFR*/*ALK* aberrations who received a first line of therapy at Frankfurt University Hospital between January 2012 and December 2018, with follow-up to December 2019 or death, whichever occurred first. Using electronic medical records, treatment patterns and survival outcomes were described by histology (squamous cell [SQ]; non-squamous cell [NSQ]/other) and time period (pre- and post-ICI approval).

**Results:**

Among eligible patients who started first-line treatment, 136 (pre-ICI) and 126 (post-ICI) had NSQ/other histology, and 32 (pre-ICI) and 38 (post-ICI) had SQ histology. Use of an ICI in the NSQ/other cohort increased from 5.9% (all second- or third-line) in the pre-ICI period to 57.1% (22.2% in first-line, including 13.5% as monotherapy and 8.7% combined with chemotherapy) in the post-ICI period. This was paralleled by a significant (*P* < 0.0001) prolongation of median (95% CI) OS from 9.4 (7.1–11.1) to 14.8 (12.7–20.5) months between the pre-ICI and post-ICI periods. A similar increase in the uptake of ICI was observed for the SQ cohort (from 3.1% pre-ICI [fourth-line] to 52.6% post-ICI [28.9% as first-line, including 15.8% as monotherapy and 13.2% combined with chemotherapy]); however, analysis of survival outcomes was limited by small group sizes.

**Conclusion:**

These real-world data complement clinical trial evidence on the effectiveness of ICIs in patients with advanced NSCLC and NSQ/other histology in Germany.

**Supplementary Information:**

The online version contains supplementary material available at 10.1186/s12890-022-02288-1.

## Background

In Germany, lung cancer is the leading cause of cancer-related death. In 2020, GLOBOCAN data for Germany estimated approximately 65,000 new cases and more than 50,000 deaths, accounting for 10% of new cancer diagnoses and 20% of total cancer deaths [1]. Non-small cell lung cancer (NSCLC), which represents 80–90% of all lung cancers worldwide, is typically diagnosed at an advanced stage, when treatment options have traditionally been limited and prognosis is poor [2–5]. The 5-year survival rates for patients diagnosed with advanced (stage IIIB) and metastatic (stage IV) NSCLC are only 10–22% and 3–6%, respectively [6].

Platinum-based chemotherapy has long been the standard-of-care for patients with advanced NSCLC and good performance status (PS) [2–4]. Over the past few decades, advances in the understanding of NSCLC tumor biology and the identification of actionable oncogenic driver mutations have led to the development and adoption of targeted therapies for subsets of patients, such as those with aberrations in genes for the epidermal growth factor receptor (EGFR), anaplastic lymphoma kinase (ALK), B—rapidly accelerated fibrosarcoma (BRAF), ROS1 receptor tyrosine kinase and/or neurotrophic tyrosine receptor kinase (NTRK) 1/2/3 [7].

More recently, immuno-oncology therapies, such as immune checkpoint inhibitors (ICIs) targeting the programmed cell death protein 1 (PD-1)/programmed death ligand 1 (PD-L1) axis have improved outcomes for patients with locally advanced or metastatic NSCLC with or without known driver mutations versus chemotherapy in clinical trials, both as monotherapy and in combination with chemotherapy [8–16]. In Germany, new therapies are reimbursed immediately after approval by the European Medicines Agency (EMA) on a 1-year temporary basis. Specific ICIs have been reimbursed and used in clinical practice since 2015 for patients with locally advanced or metastatic NSCLC without *EGFR*/*ALK* aberrations. Nivolumab, the first immuno-oncology agent to be reimbursed for NSCLC in Germany, was approved by the EMA in July 2015 and April 2016 as monotherapy for patients with locally advanced or metastatic squamous cell (SQ) carcinoma and non-squamous cell (NSQ) carcinoma, respectively, who have previously progressed on platinum-based chemotherapy [17–20]. The combination of nivolumab plus ipilimumab and two cycles of platinum-based chemotherapy was subsequently approved by the EMA in November 2020 for the first-line treatment of patients with metastatic NSCLC and no sensitizing *EGFR* mutations/*ALK* translocations [18, 21]. Pembrolizumab monotherapy was approved in January 2017 for the first-line treatment of patients with metastatic NSCLC whose tumors have high PD-L1 expression (≥ 50%) and no *EGFR* or *ALK* positive tumor mutations [17, 22], and pembrolizumab in combination with chemotherapy was approved in September 2018 and March 2019 for patients with NSQ and SQ NSCLC, respectively [23, 24].

As new drugs are introduced in routine clinical practice, it is important to assess their impact in real-world settings, outside the controlled environment of clinical trials. I-O Optimise is a multi‑country, observational research initiative utilizing real-world data sources to provide valuable insights into the evolving treatment landscape of thoracic malignancies [25]. Here, we explored the impact of ICIs on treatment patterns and survival outcomes for patients with locally advanced or metastatic NSCLC without known *EGFR* mutations/*ALK* rearrangements treated at Frankfurt University Hospital before and after the first reimbursements for ICIs for advanced NSCLC in Germany.

## Methods

### Study design and database overview

This was a retrospective cohort study utilizing electronic medical record (EMR) data, which were collected as part of routine clinical practice at Frankfurt University Hospital, a teaching hospital in the center of the Rhine Main region that treats more than 17,000 patients with cancer each year. All data are derived from EMRs and then manually coded into structured information by document specialists; these refined data are subsequently used to report to local cancer registries. Data are pseudonymized and not linked to any external data source.

The study was conducted in accordance with International Society for Pharmacoepidemiology (ISPE) Guidelines for Good Pharmacoepidemiology Practices and applicable regulatory requirements. Ethics approval was obtained following Ethics Committee review at Ethik-Kommission der Johann Wolfgang Goethe-Universität under reference number 274/18 and project number UCT-15-2020. As this was a retrospective study, informed consent was not required from patients.

### Patient population

All patients aged 18 years and older at initial NSCLC diagnosis who were diagnosed with locally advanced or metastatic disease between January 1, 2012 and December 31, 2018 were included in the study. Patients with locally advanced or metastatic disease were defined as those with incident stage IIIB/IV NSCLC or those initially diagnosed at an earlier stage (stage I–IIIA) who then progressed to stage IIIB/IV disease (the date of progression is included in the database). Study exclusion criteria were missing data on age or sex, histopathological codes indicating small cell lung cancer (i.e., International Classification of Diseases for Oncology [third edition; ICD-O-3] codes: 80413, 80423, 80433, 80443, 80453); a concomitant tumor within 5 years prior to NSCLC diagnosis, except for non-metastatic non-melanoma skin cancers or in situ benign neoplasms; treatment with a systemic anticancer therapy (SACT) within 5 years prior to the diagnosis date; and no documented treatment. For this specific analysis, only patients who received a first line of therapy at Frankfurt University Hospital, who had not tested positive for an *EGFR* or *ALK* aberration, and who had SACT treatment at Frankfurt University Hospital were included. All patients were followed from their index date (i.e., start of their first line of therapy) until the end of the study period (December 31, 2019), death, or date of last contact with the center, whichever occurred first.

The study population was subdivided based on histology (SQ or NSQ and other histologies [NSQ/other], which included “not otherwise specified” and “other specified” NSCLC) and the period of first-line therapy initiation, as follows: the period before ICI launch when ICI regimens were not available in routine clinical practice (“pre-ICI period”; NSQ/other: up to March 2015; SQ: up to June 2014) versus the period after ICI launch when ICI regimens were reimbursed in Germany for second- and later-line therapy (“post-ICI period”; NSQ/other: from April 2016; SQ: from July 2015). Patients who started their first line of therapy in the year prior to nivolumab launch (“washout period”; NSQ/other: April 2015–March 2016; SQ: July 2014–June 2015) were excluded from the analysis to minimize any potential crossover effect between time periods (i.e., patients in the pre-ICI period who received an ICI therapy after approval) and to enable assessment of the effect of ICI treatment.

The pre-ICI period captured all patients who started their first line of therapy more than 12 months prior to nivolumab approval in the second-line setting (NSQ/other: January 1, 2012 to March 31, 2015; SQ: January 1, 2012 to June 30, 2014). The post-ICI period captured all patients who started their first line of therapy following the approval of nivolumab in the second-line setting (NSQ: April 1, 2016 to December 31, 2018; SQ: July 1, 2015 to December 31, 2018). During part of the post-ICI period, the use of ICIs in the first-line setting was also captured after the reimbursement of pembrolizumab monotherapy for patients with PD-L1 ≥ 50% NSCLC in January 2017 and pembrolizumab and chemotherapy in advanced NSQ NSCLC in September 2018. However, no additional stratification to evaluate post-ICI treatment by first-line ICI launch date was possible due to the limited sample size.

### Outcomes

Patient and clinical characteristics were described at the start of their first line of treatment, which was defined in the Frankfurt University Hospital database as the first SACT received between the diagnosis of locally advanced or metastatic NSCLC and the first record of disease progression (as reported by clinicians in the data source) or death, whichever occurred first. A change in line of therapy was defined by the following two criteria: the occurrence of progression and evidence of the subsequent administration of a new SACT regimen. A subsequent line of therapy was defined as all SACTs received until there was a new record of disease progression or death. Changes in the SACT regimens due to toxicity or patient choice were not considered as a change in line of therapy. Overall survival (OS) was defined as the time in months from the index date until the date of death from any cause (calculated as the date of death minus the index date + 1, divided by 30). Patients alive at last contact were censored at that date or at the cutoff date, if earlier. Death dates were cross-checked against the regional death registry.

### Statistical analyses

Continuous variables were described using means, standard deviations (SDs), medians, first and third quartiles, and minimum and maximum values, whereas categorical variables were represented by the number and percentage of patients in each category. For categorical variables, data were masked when patient numbers for an individual category were greater than zero but less than five. All statistics included a 95% confidence interval (CI). No imputation method was used for missing data, except for dates relating to baseline demographics, where the missing date was imputed with the middle of the month, and the missing month was imputed with the middle of the year. Treatment sequencing for the first four lines of therapy is presented using Sankey diagrams; as above, data were masked when patient numbers for an individual treatment category were greater than zero but less than five. OS was calculated and depicted graphically by Kaplan–Meier curves, applying the index date for OS as the start date of the first or second line of therapy. Statistical differences in OS between pre-ICI and post-ICI periods were evaluated using a log rank test. Patients were followed from the index date until death or last contact with the center for those alive at the end of the study period.

## Results

### Patients

Overall, 386 patients met the eligibility criteria for the current analysis (Additional file 1: Fig. S1), including 168 receiving a first line of therapy in the pre-ICI period, 54 receiving a first line of therapy in the washout period, and 164 receiving a first line of therapy in the post-ICI period. Of the 332 patients across the pre-ICI and post-ICI period (i.e., excluding the washout period), 262 (78.9% [pre-ICI, n = 136; post-ICI, n = 126]) had NSQ/other histology and 70 (21.1% [pre-ICI, n = 32; post-ICI, n = 38]) had SQ histology.

Demographics and clinical characteristics for patients receiving a first line of therapy in the pre-ICI and post-ICI periods are shown in Table 1.

**Table 1:** Characteristics of patients with advanced NSCLC receiving first-line therapy at Frankfurt University Hospital pre-ICI and post-ICI

Characteristic, n (%)		Overall (N = 332)	Pre-ICI			Post-ICI		
			All histologies (n = 168)	NSQ/other (n = 136)	SQ (n = 32)	All histologies (n = 164)	NSQ/other (n = 126)	SQ (n = 38)
Age, years^a^	Median (Q1–Q3) Min–Max	64 (57–71) 25–86	63 (56–70) 25–86	62 (55–67) 25–86	64 (62–73) 37–82	65 (57–71) 41–86	64.5 (57–71) 41–86	66.5 (60–73) 47–85
Sex, n (%)	Male	190 (57.2)	97 (57.7)	75 (55.1)	22 (68.8)	93 (56.7)	68 (54.0)	25 (65.8)
Smoking status, n (%)^b^	Ex-smoker Never smoked Smoker Missing/Unknown	113 (34.0) 15 (4.5) 133 (40.1) 71 (21.4)	50 (29.8) 9 (5.4) 63 (37.5) 46 (27.4)	39 (28.7) 9 (6.6) 47 (34.6) 41 (30.1)	11 (34.4) 0 16 (50.0) 5 (15.6)	63 (38.4) 6 (3.7) 70 (42.7) 25 (15.2)	46 (36.5) 6 (4.8) 54 (42.9) 20 (15.9)	17 (44.7) 0 16 (42.1) 5 (13.2)
ECOG PS, n (%)^c^	0–1 ≥ 2 Missing/Unknown	270 (81.3) 57 (17.2) 5 (1.5)	140 (83.3) 26 (15.5) < 5	112 (82.4) 22 (16.2) < 5	28 (87.5) < 5 0	130 (79.3) 31 (18.9) < 5	102 (81.0) 21 (16.7) < 5	28 (73.7) 10 (26.3) 0
TNM stage, n (%)^b^	IA–IIIA IIIB–IVB	20 (6.0) 312 (94.0)	6 (3.6) 162 (96.4)	5 (3.7) 131 (96.3)	< 5 31 (96.9)	14 (8.5) 150 (91.5)	10 (7.9) 116 (92.1)	< 5 34 (89.5)
Histology, n (%)^b^	NSQ SQ NOS Other	237 (71.4) 70 (21.1) < 5 23 (6.9)	124 (73.8) 32 (19.0) < 5 11 (6.5)	124 (91.2) 0 < 5 11 (8.1)	0 32 (100) 0 0	113 (68.9) 38 (23.2) < 5 12 (7.3)	113 (89.7) 0 < 5 12 (9.5)	0 38 (100) 0 0
No. of metastatic organ sites^a^	Median (Q1–Q3)	1 (1–2)	1 (1–2)	1 (1–2)	1 (0–1)	1 (1–2)	1 (1–2)	1 (0–2)
Brain metastases, n (%)^a^	Yes	99 (29.8)	50 (29.8)	47 (34.6)	< 5	49 (29.9)	40 (31.7)	9 (23.7)
Liver metastases, n (%)^a^	Yes	52 (15.7)	27 (16.1)	24 (17.6)	< 5	25 (15.2)	20 (15.9)	5 (13.2)
Bone metastases, n (%)^a^	Yes	113 (34.0)	51 (30.4)	44 (32.4)	7 (21.9)	62 (37.8)	53 (42.1)	9 (23.7)
PD-L1 testing, n (%)	Not tested Positive ≥ 50% 1–49% Unknown PD-L1 level Negative (< 1%)	202 (60.8) 89 (26.8) 42 (12.7) 42 (12.7) 5 (1.5) 41 (12.3)	160 (95.2) 7 (4.2) 5 (3.0) 0 < 5 < 5	128 (94.1) 7 (5.1) 5 (3.7) 0 < 5 < 5	32 (100) 0 0 0 0 0	42 (25.6) 82 (50.0) 37 (22.6) 42 (25.6) < 5 40 (24.4)	24 (19.0) 69 (54.8) 30 (23.8) 37 (29.4) < 5 33 (26.2)	18 (47.4) 13 (34.2) 7 (18.4) 5 (13.2) < 5 7 (18.4)

Data were masked when patient numbers for an individual category were greater than zero but less than five.

^a^ At start of first-line therapy

^b^ At diagnosis

^c^ At closest date to start of first-line therapy

*ECOG PS* Eastern Cooperative Oncology Group performance status, *NOS* Not otherwise specified, *NSQ* Non-squamous cell, *PD-L1* Programmed death ligand 1, *SQ* Squamous cell, *TNM* Tumor node metastasis

The median (interquartile range) age of all patients at initiation of their first line of therapy was 64 (57–71) years and was similar between the time periods. Most patients (94.0%) were diagnosed with incident advanced or metastatic disease (tumor node metastasis [TNM] stage IIIB/IV); 6.0% of patients were initially diagnosed at an earlier stage (stage I–IIIA) and then progressed to advanced disease. The majority of patients (81.3%) had an Eastern Cooperative Oncology Group (ECOG) PS of 0–1 at the start of their first line of therapy. Characteristics of patients with NSQ/other histology were generally similar between the pre-ICI and post-ICI periods, although those treated in the post-ICI period tended to be slightly older and have a less advanced stage at diagnosis. Among patients with SQ histology, those in the post-ICI period tended to be slightly older, had a higher ECOG PS, more frequently had brain metastases at the start of their first line of therapy, and tended to have less advanced stage at diagnosis compared with those in the pre-ICI period; however, the overall number of patients with SQ histology was low in both time periods. As expected, PD-L1 testing increased between the pre-ICI and post-ICI periods, with 5.9% and 0.0% of patients with NSQ/other and SQ histology, respectively, undergoing PD-L1 testing in the pre-ICI period versus 81.0% and 52.6% in the post-ICI period.

### Treatment patterns by time period and histology

#### First line of treatment

**Fig. 1:**
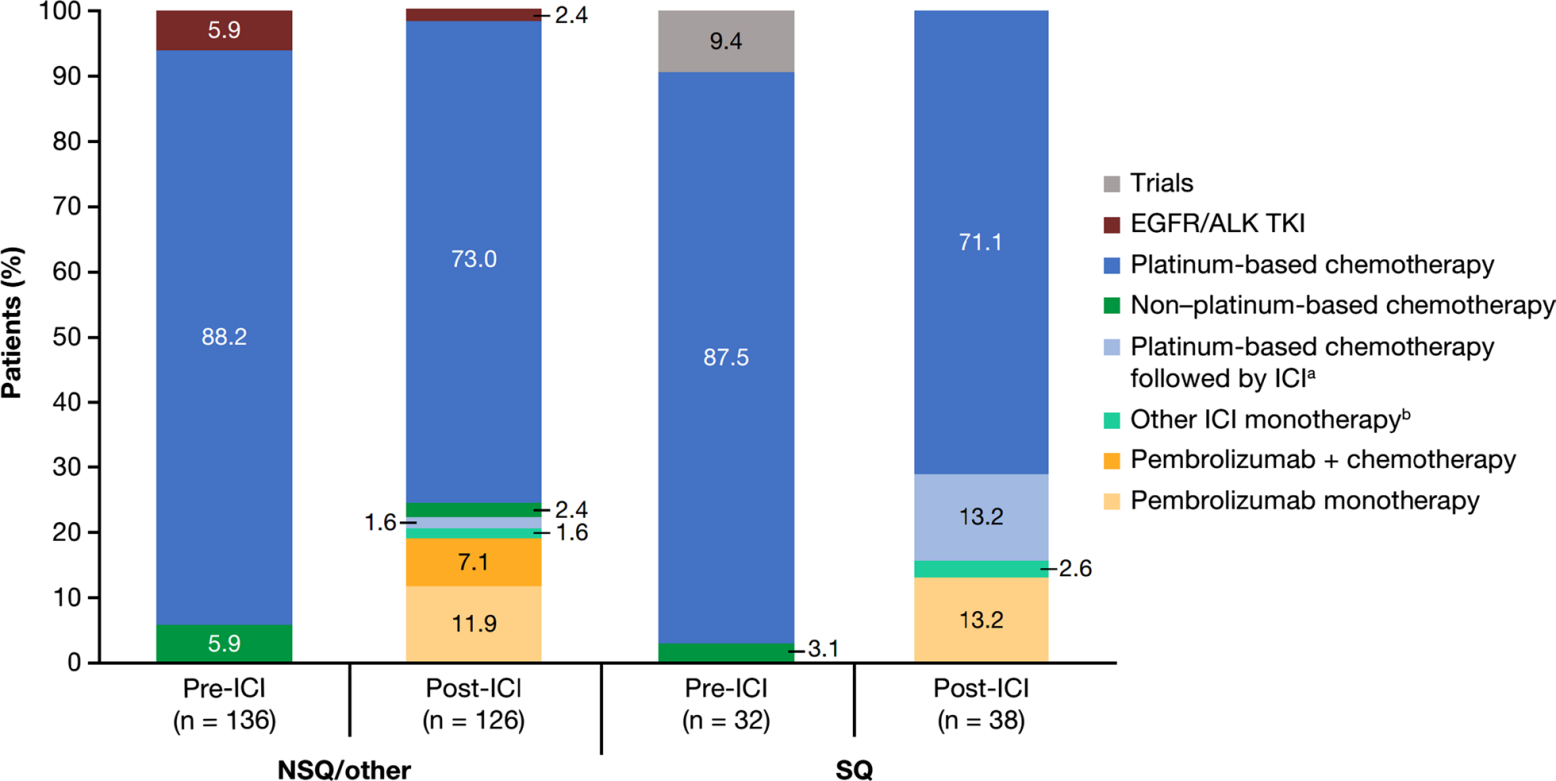
First-line treatment regimens received in the pre-ICI and post-ICI periods by histology. ^a^ Patients who discontinued a platinum-based chemotherapy and initiated an ICI other than pembrolizumab without any progression reported prior to ICI start. Under these circumstances, the ICI was considered as part of the first-line regimen. ^b^ ICI monotherapy other than pembrolizumab (i.e., not indicated in first line); patients in this category were likely to have been refractory to a previous multimodal treatment administered for non-metastatic disease. *ALK* Anaplastic lymphoma kinase, *EGFR* Epidermal growth factor receptor, *ICI* Immune checkpoint inhibitor; *NSQ* Non-squamous cell, *SQ* Squamous cell, *TKI* Tyrosine kinase inhibitor

In the pre-ICI period, most patients with NSQ/other histology (88.2%) or SQ histology (87.5%) received platinum-based chemotherapy as first line of therapy, declining to 73.0% and 71.1% in the post-ICI period, respectively (Fig. 1). As expected, no patients received an ICI in the first-line setting, either as monotherapy or in combination with chemotherapy, in the pre-ICI period. In the post-ICI period, 22.2% of patients with NSQ/other histology and 28.9% of patients with SQ histology received an ICI in the first-line setting, either as monotherapy or in combination with chemotherapy. Overall, 11.9% of patients with NSQ/other histology and 13.2% with SQ histology received pembrolizumab monotherapy in the first-line setting, while 7.1% of patients with NSQ/other histology received pembrolizumab in combination with chemotherapy.

#### Second line of treatment

**Fig. 2:**
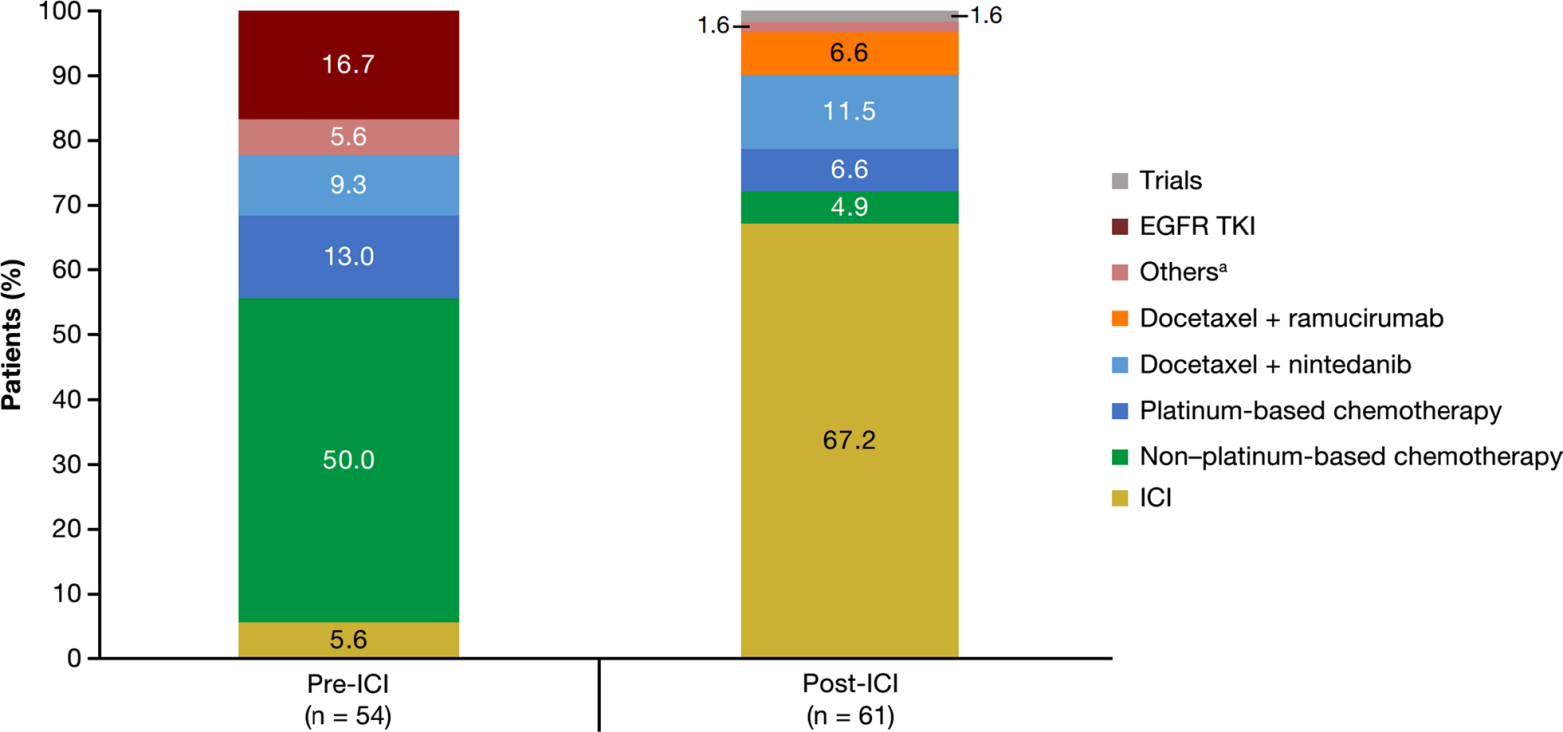
Second-line treatment regimens received by patients with NSQ/other histology in the pre-ICI and post-ICI periods. ^a^ Other treatments included cabozantinib (n = 1) and capmatinib (n = 2) in the pre-ICI period and dabrafenib plus trametinib (n = 1) in the post-ICI period. *EGFR* Epidermal growth factor receptor, *ICI* Immune checkpoint inhibitor, *NSQ* Non-squamous cell, *TKI* Tyrosine kinase inhibitor

The characteristics of patients with NSQ/other histology who received a second line of therapy in the pre-ICI and post-ICI periods are shown in Additional file 1: Table S1. The proportion of patients with NSQ/other histology who received a second line of therapy increased slightly between the pre-ICI and post-ICI periods, from 39.7 to 48.4%, respectively (Additional file 1: Fig. S2). Of these patients, the proportion receiving an ICI increased from 5.6% in the pre-ICI period to 67.2% in the post-ICI period (Fig. 2). In parallel, the proportion of patients who received non-platinum-based chemotherapy or tyrosine kinase inhibitor (TKI) monotherapy as their second line of therapy decreased from 50.0% and 16.7% in the pre-ICI period to 4.9% and 0.0%, respectively, in the post-ICI period.

There was no change in the proportion of patients with SQ histology who received a second-line therapy over the study period (31.3% [10/32] in the pre-ICI period versus 31.6% [12/38] in the post-ICI period) (Additional file 1: Fig. S2). Consistent with observations in the NSQ/other histology cohort, the proportion of patients with SQ histology receiving non-platinum-based chemotherapy as their second line of therapy decreased between the pre-ICI (50.0%) and post-ICI (8.3%) periods, concomitant with a notable increase in the use of ICIs in this second-line setting: from 0.0% (0/10) in the pre-ICI period to 66.7% (8/12) in the post-ICI period. However, the overall number of patients was consistently low.

The overall share of patients with NSQ/other histology who received an ICI either as monotherapy or in combination with chemotherapy across the course of their treatment (i.e., across four lines of therapy) increased from 5.9% (all second- or third-line) in the pre-ICI period to 57.1% (22.2% in first-line and 34.9% in second- or third-line) in the post-ICI period, while the respective proportions for patients with SQ histology were 3.1% (fourth-line) and 52.6% (28.9% in first-line and 23.7% in second- or third-line) (Additional file 1: Figs. S3 and S4). Overall trends in treatment sequencing patterns for patients with NSQ/other and SQ histology are shown across the first four lines of therapy in Additional file 1: Fig. S4.

### Overall survival

The median follow-up (Q1–Q3) from the start of first line of therapy until death or last contact with the center was 8.8 (3.4–15.5) months in the pre-ICI period and 12.8 (4.1–22.2) months in the post-ICI period for patients with NSQ/other histology and 11.5 (6.4–18.3) months in the pre-ICI period and 10.6 (6.1–15.7) months in the post-ICI period for patients with SQ histology.

**Fig. 3:**
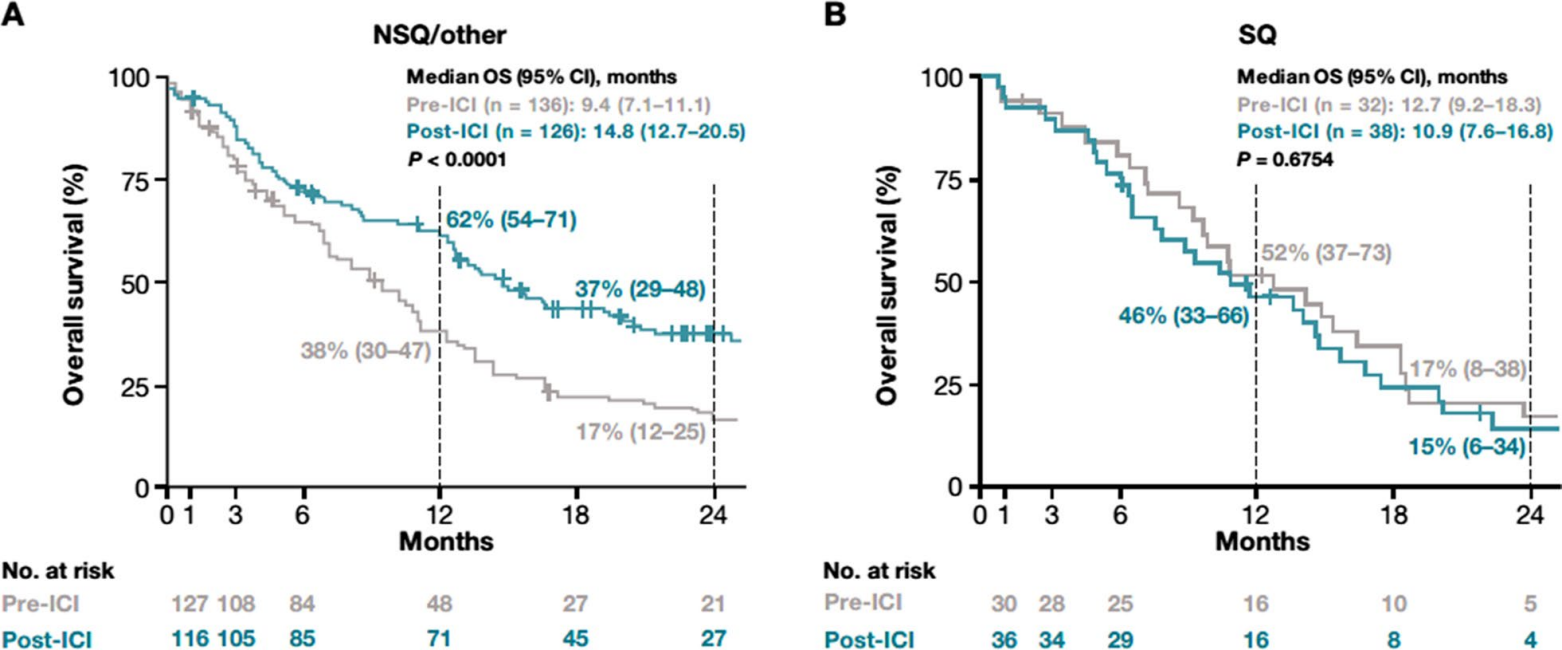
Overall survival for patients with NSQ/other (**A**) or SQ (**B**) histology receiving a first line of therapy in the pre-ICI and post-ICI periods. Index date represents the start of first-line therapy. *CI* Confidence interval, *ICI* Immune checkpoint inhibitor, *NSQ* Non-squamous cell, *OS* Overall survival, *SQ* Squamous cell

As shown in Fig. 3, median (95% CI) OS for patients with NSQ/other histology who received a first line of therapy increased significantly from 9.4 (7.1–11.1) months in the pre-ICI period to 14.8 (12.7–20.5) months in the post-ICI period (*P* < 0.0001). One-year OS rates were 38% (30–47%) versus 62% (54–71%) in the pre-ICI and post-ICI periods, respectively; 2-year OS rates were 17% (12–25%) versus 37% (29–48%), respectively.

**Fig. 4:**
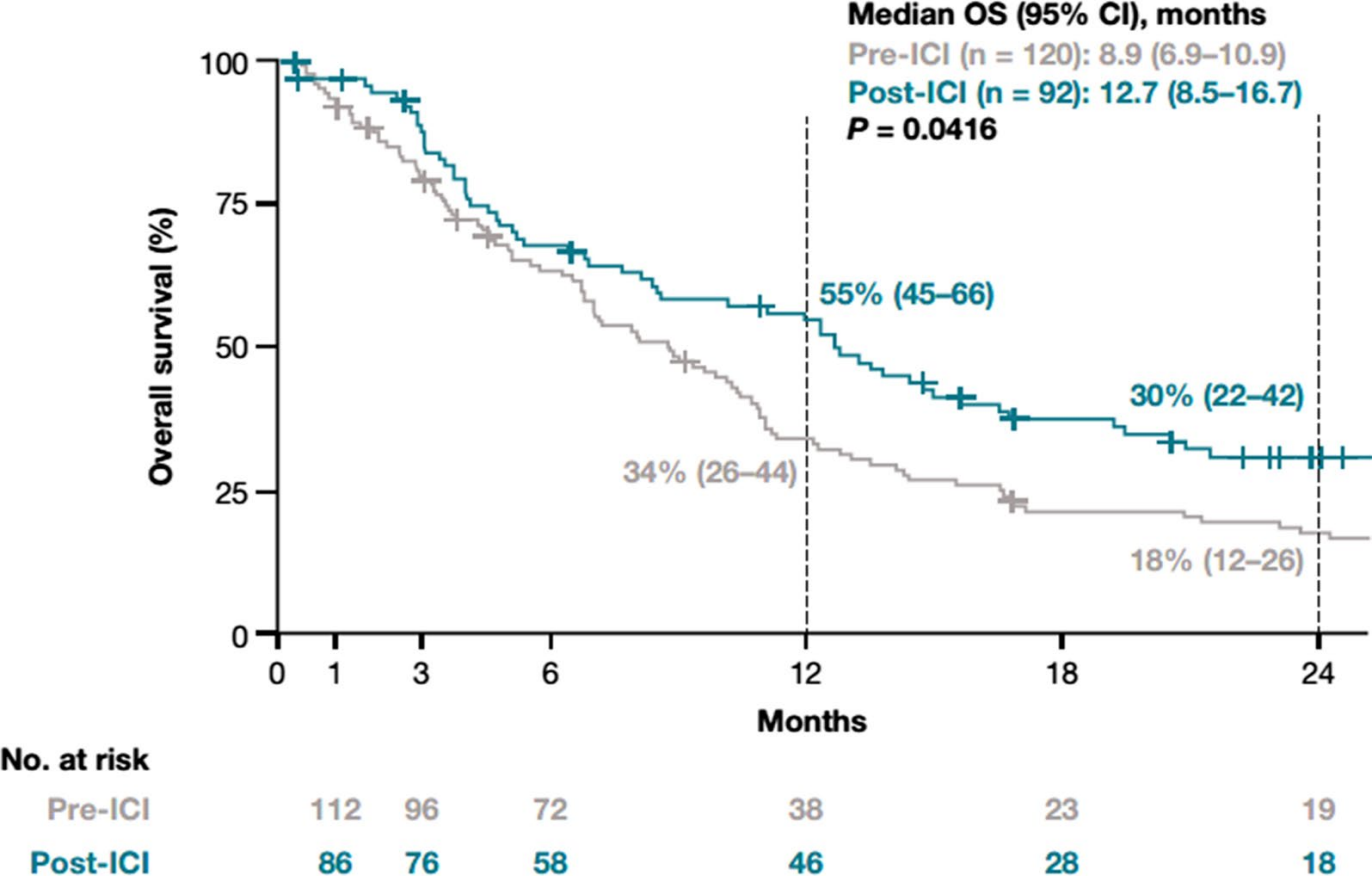
Overall survival for patients with NSQ/other histology receiving first-line platinum-based therapy in the pre-ICI and post-ICI periods. Includes only those patients receiving a first-line platinum-based chemotherapy regimen alone (i.e., no ICI therapy in first-line setting). Index date represents the start of first-line therapy. *CI* Confidence interval, *ICI* Immune checkpoint inhibitor, *NSQ* Non-squamous cell, *OS* Overall survival

Among patients with NSQ/other histology who received a first-line regimen containing platinum-based chemotherapy (i.e., may have been eligible for second-line treatment with an ICI), median OS from the initiation of first-line treatment increased from 8.9 (6.9–10.9) months in the pre-ICI period to 12.7 (8.5–16.7) months in the post-ICI period (*P* = 0.0416). Corresponding 1-year OS rates in the pre- and post-ICI periods were 34% (26–44%) and 55% (45–66%), respectively, and the 2-year OS rates were 18% (12–26%) and 30% (22–42%), respectively (Fig. 4).

**Fig. 5:**
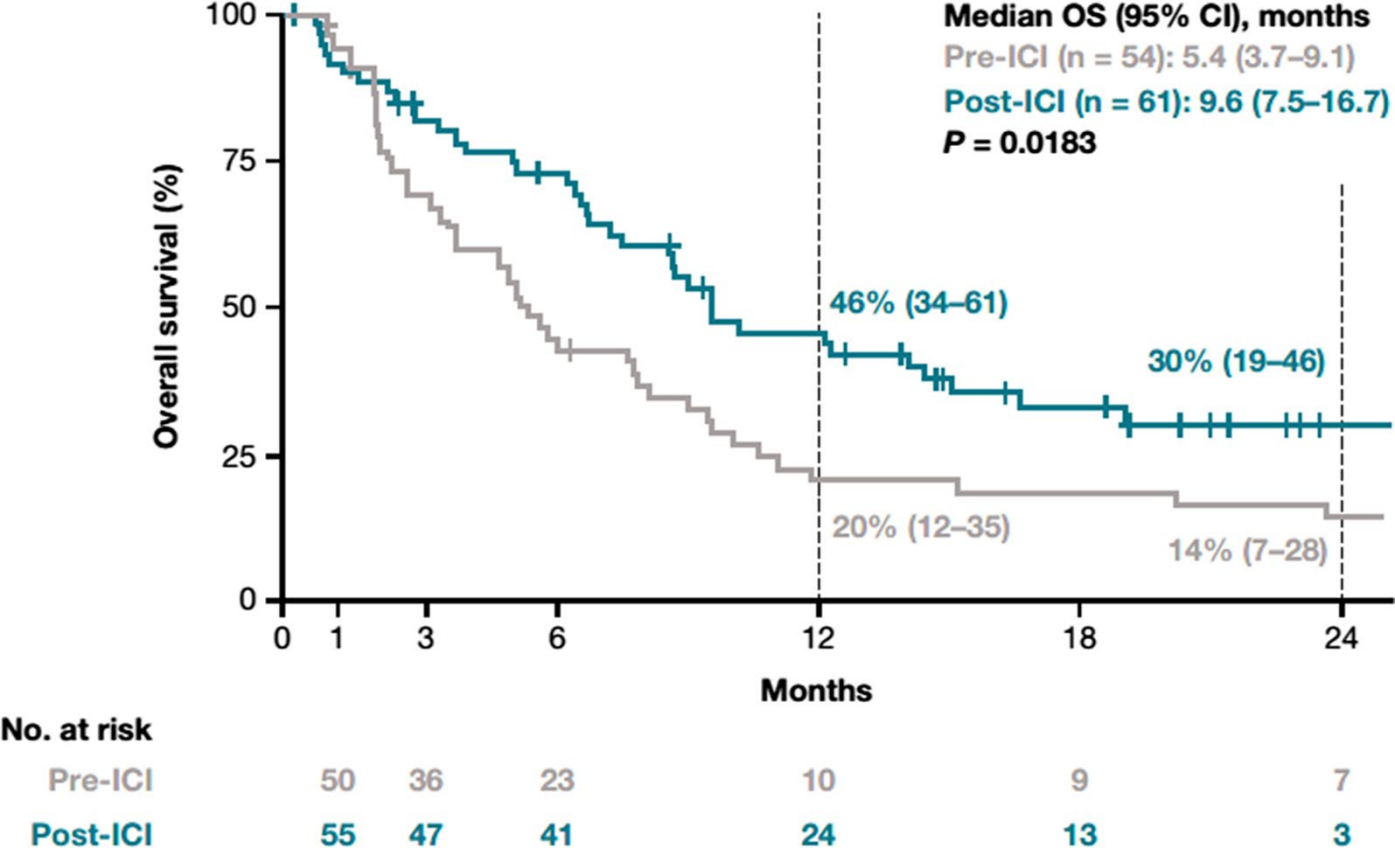
Overall survival for patients with NSQ/other histology receiving a second line of therapy in the pre-ICI and post-ICI periods. Index date represents the start of second-line therapy. *CI* Confidence interval, *ICI* Immune checkpoint inhibitor, *NSQ* Non-squamous cell, *OS* Overall survival

In patients with NSQ/other histology who received a second line of therapy, median OS was 5.4 (3.7–9.1) months in the pre-ICI period and 9.6 (7.5–16.7) months in the post-ICI period (*P* = 0.0183). One-year OS rates following second-line therapy were 20% (12–35%) and 46% (34–61%) in the pre-ICI and post-ICI periods, respectively (Fig. 5).

Among patients with SQ histology, median (95% CI) OS for those who received a first line of therapy was 12.7 (9.2–18.3) months in the pre-ICI period and 10.9 (7.6–16.8) months in the post-ICI period (*P* = 0.6754) (Fig. 3). No further analyses were performed on the SQ cohorts, as only 10 and 12 patients in the pre-ICI and post-ICI periods, respectively, received a second line of therapy.

## Discussion

Using data extracted from EMRs at Frankfurt University Hospital, our study showed that ICIs were quickly adopted in clinical practice following their first approval for NSCLC in 2015. Notably, the overall share of patients who received an ICI over the course of their treatment increased by around 50% following the reimbursement of these therapies for NSCLC in Germany. Furthermore, in parallel with this adoption of ICIs, there was a meaningful survival improvement among the cohort of patients with NSQ/other histology, with the median OS for these patients increasing by almost 6 months over the study period. The rapid adoption of ICIs observed in this single center study from Germany supports observations in larger data sets from the UK [26], Canada [27], and the US [28–33]. Notwithstanding this finding, only 57% of patients with NSQ/other histology and 53% of patients with SQ histology received an ICI in the post-ICI period of this study. As such, additional analyses will be necessary to assess future ICI uptake, which is expected to further increase with wider access to first-line ICI and chemotherapy combinations following their first approval in September 2018.

Consistent with contemporaneous guideline recommendations [3, 4] and data from other real-world studies in Germany [34], Europe [35, 36], Canada [27], and the US [37], most patients in our study population received first-line treatment with a platinum-based chemotherapy regimen. In the post-ICI period, we also observed a small proportion of patients receiving pembrolizumab monotherapy in the first-line setting (11.9% and 13.2% of patients with NSQ/other and SQ histology, respectively), subsequent to its approval in high PD-L1 expressors (≥ 50%) in January 2017. Similarly, a small proportion of patients with NSQ/other histology (7.1%) received first-line pembrolizumab plus chemotherapy following the approval of this regimen in September 2018 (which was close to the end of the inclusion period [December 2018]). Nevertheless, due to the period captured in our study, the largest increase in the proportion of patients receiving ICIs during the post-ICI period was among those prescribed these agents in the second- or later-line settings.

Notably, the overall proportion of patients with NSQ/other histology who received a second line of therapy at Frankfurt University Hospital increased from 39.7% to 48.4% between the pre-ICI and post-ICI periods, possibly due to the availability of new and well-tolerated treatment options in this setting. Real-world evidence from the Flatiron Health database found a similar increase in the proportion of patients who received a second-line therapy following the approval of ICIs in the US (from 37% pre-ICI to 57.0% post-ICI) [30]. Among patients with SQ histology, only around one-third of patients received a second line of therapy, with no difference observed between the pre-ICI and post-ICI periods. Small patient numbers in the SQ cohorts may underlie the lack of observed difference between the two periods. Half of all patients who received a second-line therapy in the pre-ICI period were treated with non-platinum-based chemotherapy; however, in the post-ICI period, two-thirds of patients received an ICI in this setting (67.2% and 66.7% for patients with NSQ/other and SQ histology, respectively). These treatment patterns are consistent with European Society for Medical Oncology (ESMO) guidelines at the time of the study [3, 4] and with current international practice guidelines recommending an ICI for patients without actionable driver mutations [2, 38]. Contrary to earlier guidance [3, 4], a notable proportion of patients with NSQ/other histology who did not have known *EGFR*/*ALK* aberrations received EGFR/ALK-targeted therapy as first-line therapy (5.9%; crizotinib, erlotinib, or afatinib) and EGFR-targeted therapy as second-line therapy (16.7%; all erlotinib) in the pre-ICI period, suggesting a measure of non-adherence to guideline recommendations in clinical practice at the time. However, this observation should be interpreted cautiously as some of these patients may have had a *ROS* mutation and patients with *ROS* mutations were not excluded from this study. Interestingly, this observation is similar to data from the multinational PIvOTAL study, in which a TKI was received by up to 11% of patients without a confirmed *EGFR* mutation/*ALK* rearrangement in the first-line setting and in 9–56% of patients in the second-line setting [35], and  aligns with studies in Germany (CRISP registry) and the US (Flatiron database) showing that only around three-quarters of patients with advanced or metastatic NSCLC undergo *EGFR* testing prior to initiation of a first-line TKI [39, 40].

The observed increased adoption of ICIs in the NSQ/other cohort was paralleled by significant survival improvements, with the median OS prolonged by 5.4 months between the pre-ICI and post-ICI period and increased OS rates at both the 1-year (from 38% to 62%) and 2-year (from 17% to 37%) landmarks. Although this study was not designed to specifically attribute OS improvements to certain treatment options, on the basis of clinical trials showing improved survival outcomes with ICIs compared with chemotherapy in both the first-line [11, 12] and second-line [8–10, 13] settings, it is likely that the observed OS improvements were at least partly due to the uptake of ICIs in the post-ICI period. While the follow-up time of our study is not yet sufficient to evaluate the impact of first-line ICIs on survival, the improvement in OS in the NSQ/other cohort for patients who received a platinum-based regimen in the first-line setting (i.e., were eligible to receive an ICI in second line) is consistent with the assumption that the observed survival improvement was mainly related to the use of ICIs in the second-line setting. This conclusion is supported by recent analyses based on US databases (Surveillance, Epidemiology, and End Results [SEER] and Flatiron), which found that survival following an NSCLC diagnosis increased in the US when novel therapeutic agents, including ICIs, became available [30, 41]. This was expanded upon by a temporal analysis of data from EMRs, which found that the risk of death was 21% lower for patients with advanced/metastatic NSCLC without documented oncogenic drivers diagnosed in 2019 versus those diagnosed in 2012. These findings support the notion that changes in treatment patterns underlie the temporal improvements in OS observed since ICIs have been widely available in the US [42].

Despite the increased ICI uptake in the SQ cohort, no significant improvements in OS were observed between the pre-ICI and post-ICI periods (median of 12.7 months versus 10.9 months, respectively; *P* = 0.6754). The comparative differences between the NSQ/other and SQ cohorts are consistent with clinical trial and real-world findings of a trend for lower survival gains in patients with SQ histology receiving ICIs [13, 29]. The lack of a significant OS improvement in the SQ cohort may also reflect, in part, differences in certain baseline characteristics between patients treated in the pre-ICI and post-ICI periods. Indeed, a noticeably larger proportion of patients with SQ histology treated in the post-ICI period had an ECOG PS ≥ 2 and/or brain metastases, characteristics that have been previously shown to potentially impact OS in treated patients with advanced NSCLC [43–48]. Moreover, the small number of patients in the overall SQ cohort (32 and 38 in the pre-ICI and post-ICI periods, respectively), as well as the low proportion treated in second line (representing only 10 and 12 patients in the pre-ICI and post-ICI periods, respectively) severely limited analysis of OS outcomes in this population and these results should be interpreted with caution.

### Strengths and limitations

The main strength of this observational study lies in the fact that the Frankfurt University Hospital data source facilitated an insight into the real-world treatment of patients with locally advanced or metastatic NSCLC in Frankfurt using appropriate quality control measures to limit inconsistency in the collected data. The ability to extract treatment outcomes directly from the data rather than extrapolating from administered therapies and treatment intervals presents an additional strength of the study. Furthermore, for those patients who died in the region of Hesse, death dates were cross-checked against a regional registry. Notably, the exclusion of patients with known *EGFR*/*ALK* aberrations in this analysis ensured that the patient population was representative of those who would be considered eligible to receive an ICI in the second-line setting following their reimbursement.

Study limitations include those inherent to the design of retrospective EMR-derived data analyses, such as the potential for miscoding and missing data values [49]. It should also be noted that data demonstrating the rapid adoption of ICIs reported herein were drawn from a study population treated in a large academic center with potentially greater access to, and more experience of, emerging therapies compared with other treatment centers in Germany. Therefore, the evolution of treatment patterns reported herein may not be representative of the treatment landscape at a national level. Additional constraints related to limited sample size and follow-up time mean that it was not possible to determine the impact of ICI therapies in patients with SQ in our study. Finally, it should be acknowledged that general advances in disease management and palliative care approaches over the course of the study could have contributed to the observed OS improvements in the NSQ/other cohort.

## Conclusion

Real-world databases, such as registries and EMRs, if adequately maintained, can provide rapidly accessible insights into clinical practice, which can be used to complement data obtained from clinical trials. The real-world results reported herein support clinical trial data on the effectiveness of ICI therapies for patients with advanced NSCLC with NSQ/other histology in clinical practice in Germany. A larger sample size and longer follow-up are needed to better understand the impact of ICI therapies in patients with SQ histology.

## Supplementary Information

**Additional file 1: Table S1.** Characteristics of patients with advanced NSCLC and NSQ/other histology receiving second line therapy in the pre-ICI and post-ICI periods.** Fig. S1.** Patient flow chart. **Fig. S2.** Proportions of patients with NSQ/other (A) or SQ (B) histology receiving second line therapy in the pre-ICI and post-ICI periods.** Fig. S3.** Proportions of patients receiving an ICI across any of their first four lines of therapy in the pre-ICI and post-ICI periods by histology.** Fig. S4.** Treatment sequencing in the pre-ICI and post-ICI periods by histology

## Data Availability

The data from this study are not publicly available and no data sharing is planned. Patient level data cannot to be shared due to regulatory and confidentiality reasons. Aggregate results from the study are presented in this manuscript. Further questions on data sharing should be directed to the corresponding author (Dr Andrea Wolf).

## Abbreviations

ALK: Anaplastic lymphoma kinase
BRAF: B—rapidly accelerated fibrosarcoma
CI: Confidence interval
ECOG: Eastern Cooperative Oncology Group
EGFR: Epidermal growth factor receptor
EMA: European Medicines Agency
EMR: Electronic medical record
ICI: Immune-checkpoint inhibitor
ISPE: International Society for Pharmacoepidemiology
NOS: Not otherwise specified
NSCLC: Non-small cell lung cancer
NSQ: Non-squamous cell
NTRK: Neurotrophic tyrosine receptor kinase
OS: Overall survival
PD-1: Programmed cell death protein 1
PD-L1: Programmed death ligand 1
PS: Performance status
SACT: Systemic anticancer therapy
SD: Standard deviation
SQ: Squamous cell
TKI: Tyrosine kinase inhibitor
TNM: Tumor node metastasis
