# Supplementary material for: Evolution of treatment patterns and survival outcomes in patients with advanced non-small cell lung cancer treated at Frankfurt University Hospital in 2012–2018
Source: BMC Pulm Med. 2023 Jan 13;23:16. doi: 10.1186/s12890-022-02288-1 (PMC9838033; doi:10.1186/s12890-022-02288-1)
Supplement: Supplementary file 1 — Additional file 1: Table S1. Characteristics of patients with advanced NSCLC and NSQ/other histology receiving second line therapy in the pre-ICI and post-ICI periods. Fig. S1. Patient flow chart. Fig. S2. Proportions of patients with NSQ/other (A) or SQ (B) histology receiving second line therapy in the pre-ICI and post-ICI periods. Fig. S3. Proportions of patients receiving an ICI across any of their first four lines of therapy in the pre-ICI and post-ICI periods by histology. Fig. S4. Treatment sequencing in the pre-ICI and post-ICI periods by histology [file 12890_2022_2288_MOESM1_ESM.docx]

**ADDITIONAL FILE 1**

**Evolution of treatment patterns and survival outcomes in patients with advanced non-small cell lung cancer treated at Frankfurt University Hospital in 2012–2018**

Andrea Wolf, et al.

**Table S1 Characteristics of patients with advanced NSCLC and NSQ/other histology** receiving second line therapy **in the pre-ICI and post-ICI periods**

| Characteristic | | Pre-ICI  (n = 54) | Post-ICI  (n = 61) |
| --- | --- | --- | --- |
| Age, years^a^ | Median (Q1–Q3)  Min–max | 63 (56–68)  26–85 | 66 (60–70)  41–86 |
| Sex, n (%) | Male | 24 (44.4) | 36 (59.0) |
| Smoking status, n (%)^b^ | Ex-smoker  Never smoked  Smoker  Missing/unknown | 21 (38.9)  < 5  17 (31.5)  13 (24.1) | 26 (42.6)  < 5  26 (42.6)  6 (9.8) |
| ECOG PS, n (%)^c^ | 0–1  ≥ 2 | 44 (81.5)  10 (18.5) | 57 (93.4)  < 5 |
| TNM stage, n (%)^b^ | IA–IIIA  IIIB–IVB | 0  54 (100) | 5 (8.2)  56 (91.8) |
| Histology, n (%)^b^ | NSQ  NOS  Other | 50 (92.6)  0  < 5 | 54 (88.5)  0  7 (11.5) |
| Brain metastases, n (%)^a^ | Yes | 21 (38.9) | 24 (39.3) |
| PD-L1 testing, n (%) | Not tested  Positive  ≥ 50%  1–49%  Unknown PD-L1 level  Negative (< 1%) | 46 (85.2)  7 (13.0)  5 (9.3)  0  < 5  < 5 | 9 (14.8)  33 (54.1)  14 (23.0)  18 (29.5)  < 5 19 (31.1) |

**Data were masked when patient numbers for an individual category were greater than zero but less than five**

**^a^ At start of second-line therapy**

**^b^ At diagnosis**

**^c^ At closest date to start of second-line therapy**

***ECOG PS* Eastern Cooperative Oncology Group performance status, *NOS* not otherwise specified,
*NSQ* non-squamous cell, *PD-L1* programmed death ligand 1, *SQ* squamous cell, *TNM* tumor node metastasis**

**Fig. S1** Patient flow chart


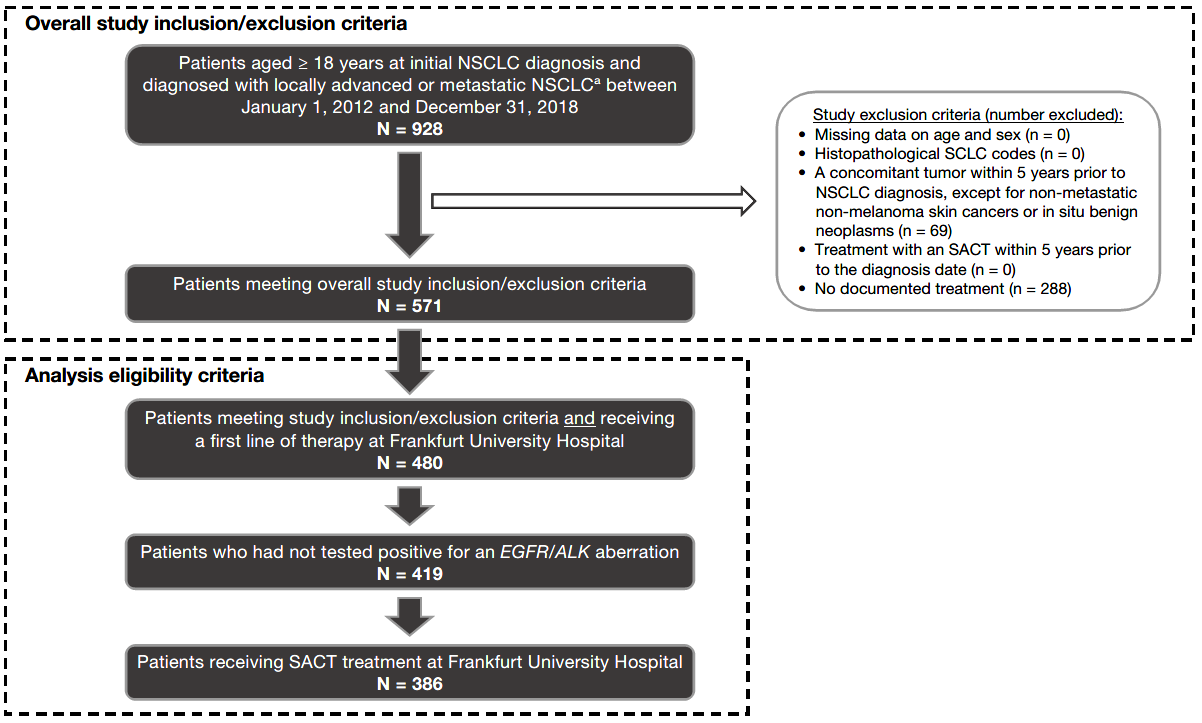


**^a^ Incident stage IIIB/IV NSCLC or progressed stage I-IIIA NSCLC (with progression at Frankfurt University Hospital)**

***SACT* systemic anticancer therapy, *SCLC* small cell lung cancer**

**Fig. S2** Proportions of patients with NSQ/other (**A**) or SQ (**B**) histology receiving second line therapy **in the pre-ICI and post-ICI periods**
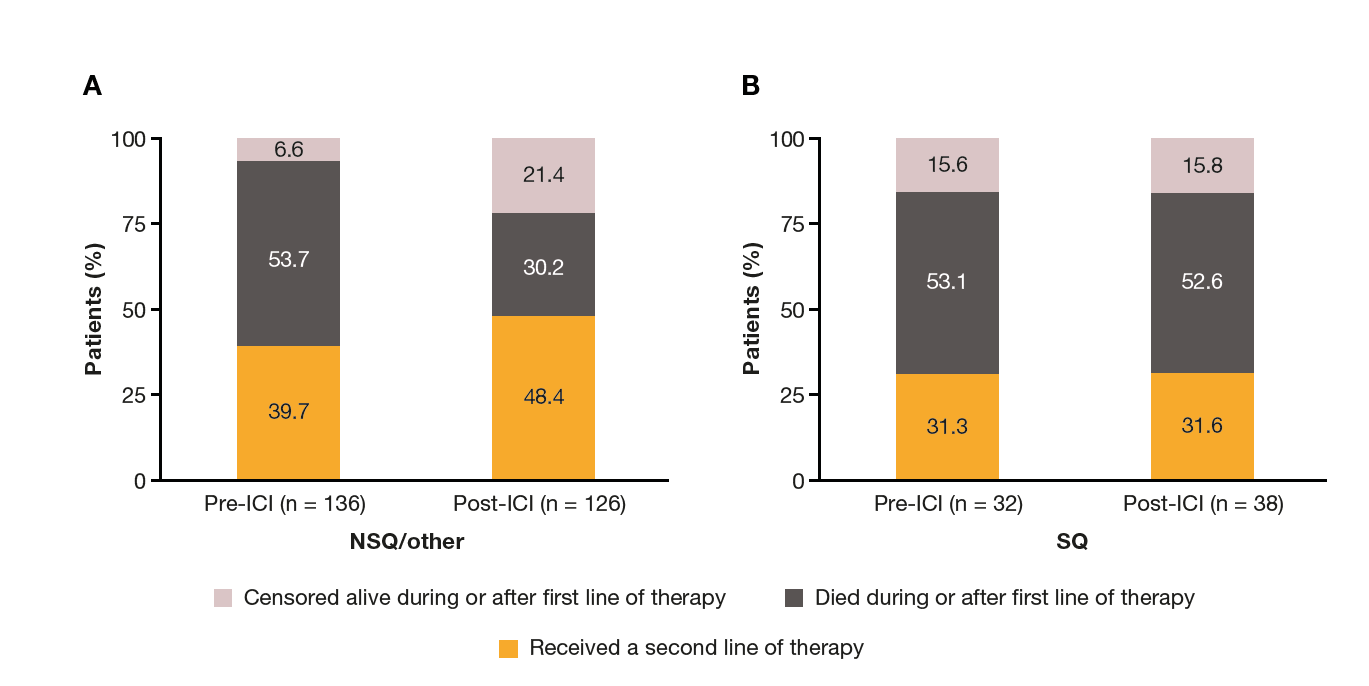


***ICI* immune checkpoint inhibitor, *NSQ* non-squamous cell, *SQ* squamous cell**

**Fig. S3** Proportions of patients receiving an ICI across any of their first four lines of therapy in the pre-ICI and post-ICI periods by histology


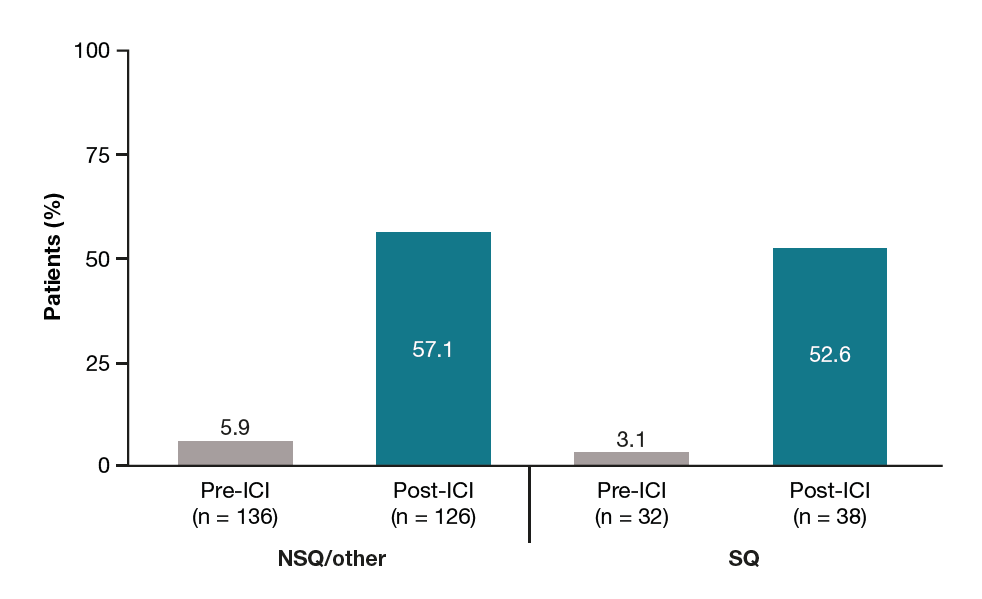


*ICI* immune checkpoint inhibitor**, *NSQ* non-squamous cell, *SQ* squamous cell**

**Fig. S4 Treatment sequencing** in the pre-ICI and post-ICI periods by histology
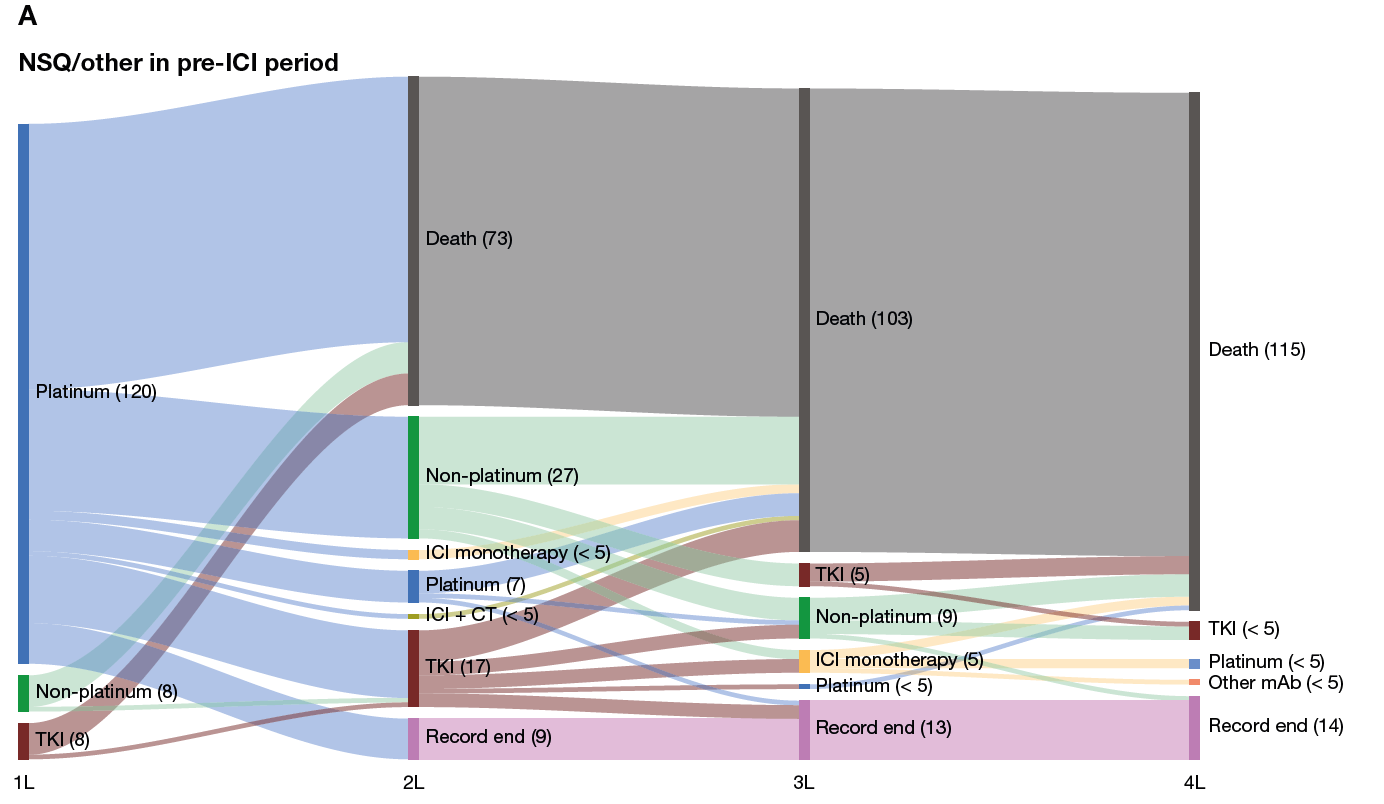


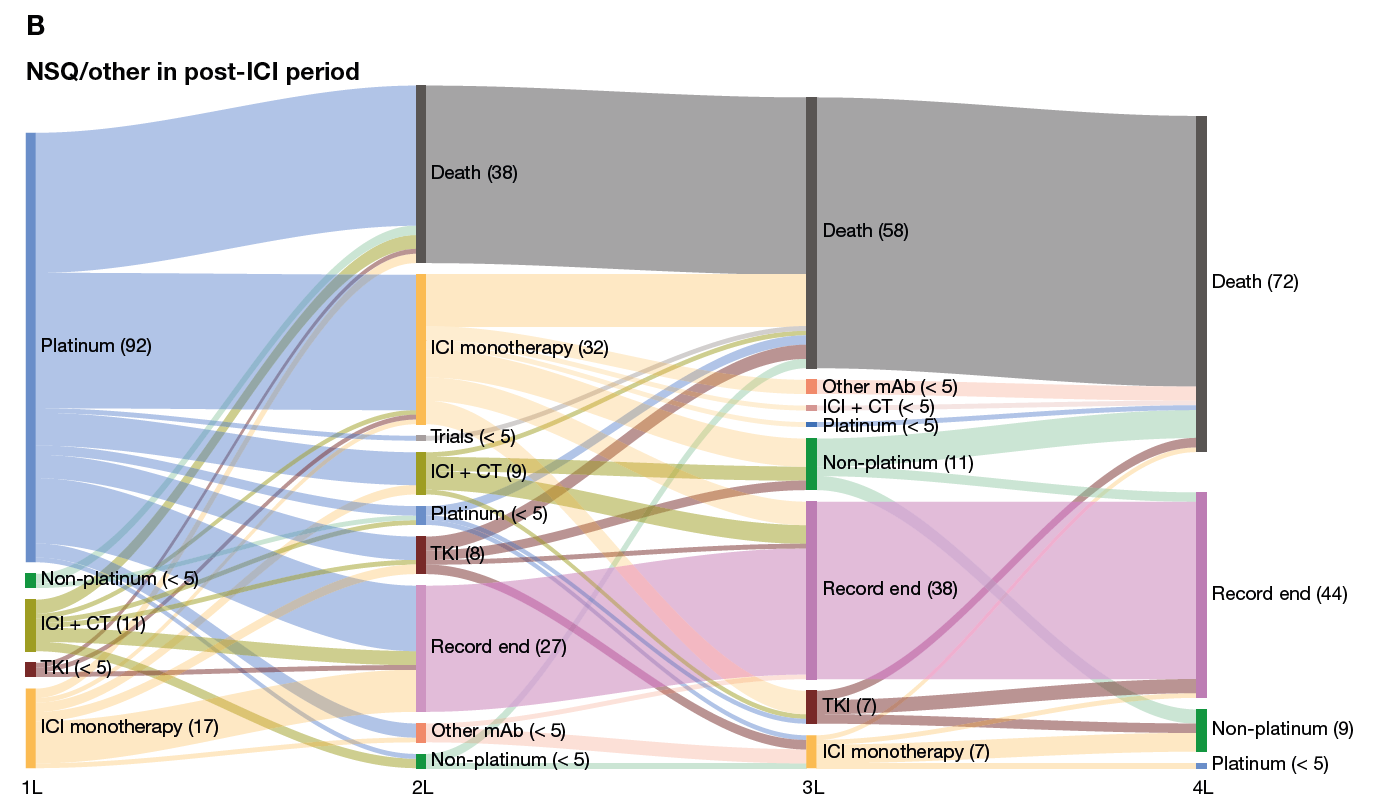


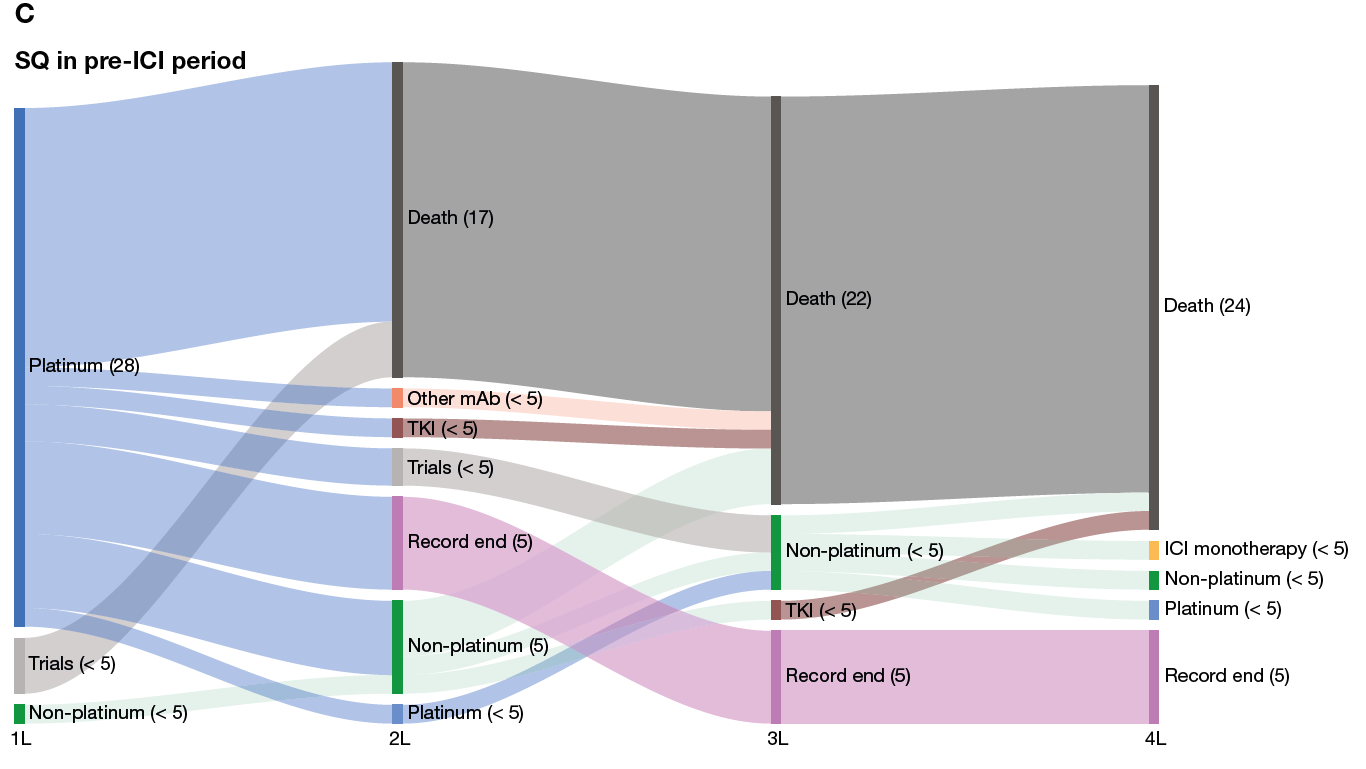


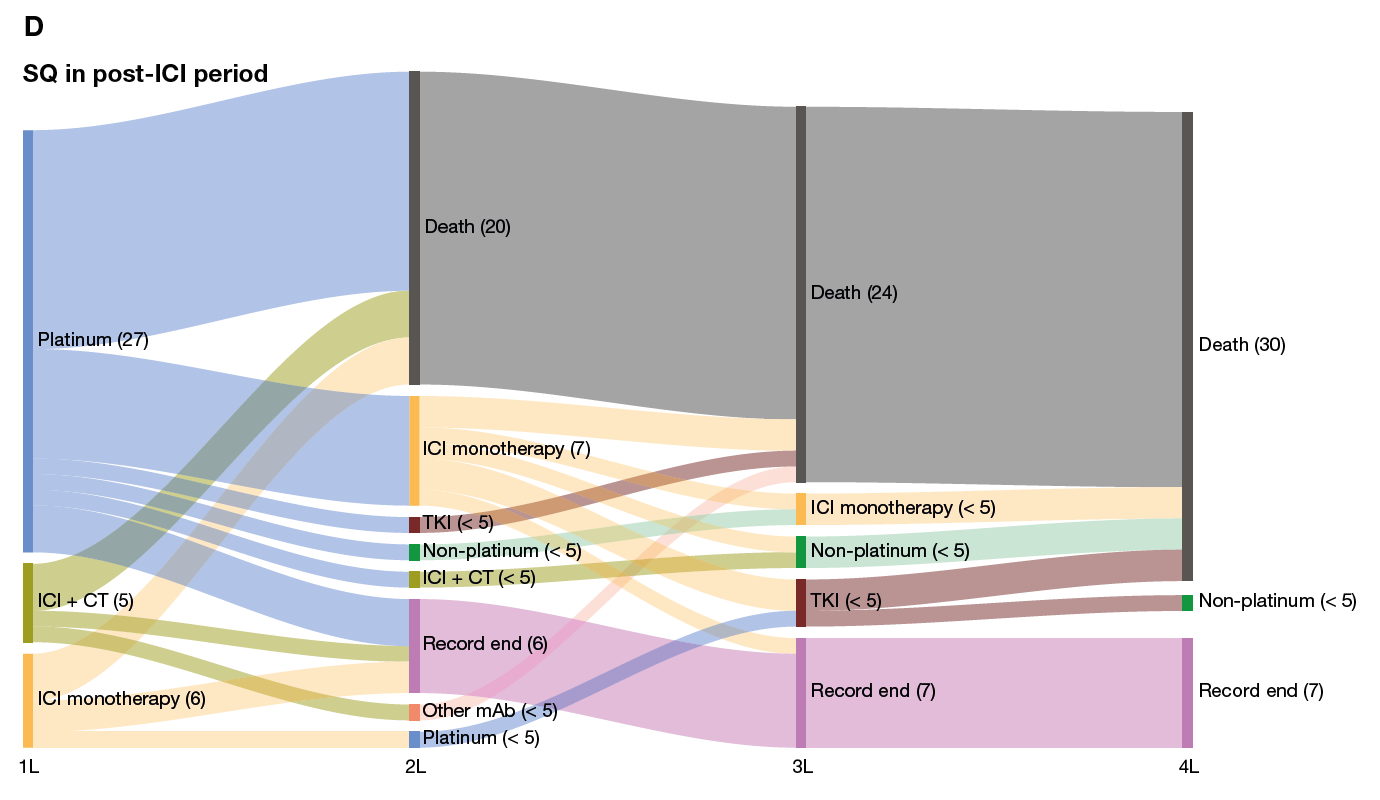


**Data were masked when patient numbers for an individual category were greater than zero but less than five
*1L* first line, *2L* second line, *3L* third line, *4L* fourth line, *CT* chemotherapy, *ICI* immune checkpoint inhibitor, *mAb* monoclonal antibody, *NSQ* non-squamous cell,
*SQ* squamous cell, *TKI* tyrosine kinase inhibitor**
